# Knowledge, attitude and practice towards insulin self-administration and associated factors among diabetic patients at Zewditu Memorial Hospital, Ethiopia

**DOI:** 10.1371/journal.pone.0246741

**Published:** 2021-02-08

**Authors:** Beshir Bedru Nasir, Miftah Shafi Buseir, Oumer Sada Muhammed

**Affiliations:** 1 Department of Pharmacology and Clinical Pharmacy, School of Pharmacy, College of Health Sciences, Addis Ababa University, Addis Ababa, Ethiopia; 2 Department of Pharmacy, College of Health Sciences, Mizan-Tepi University, Tepi, Ethiopia; University of the Witwatersrand, SOUTH AFRICA

## Abstract

**Background:**

Diabetes mellitus is a common health problem worldwide. Proper insulin administration plays an important role in long term optimal blood sugar control. Adequate knowledge and attitude about insulin self-administration could also improve the management of diabetes and eventually improve the quality of life. This study aimed to assess knowledge, attitude and practice towards insulin self-administration and associated factors among diabetic patients at Zewditu Memorial Hospital (ZMH), Ethiopia.

**Methods:**

An institution-based cross-sectional study was conducted among 245 diabetic patients who were selected by systematic random sampling during follow-up at ZMH. The data was collected using an interviewer-administered structured questionnaire and analyzed by SPSS v.20. Binary logistic regression was used to identify associated factors of patients’ knowledge and *P < 0*.*05* was used to declare the association.

**Results:**

Among 245 patients enrolled, 53.9% were male with a mean age of 53.26 ±13.43 years and more than 84% of the patients can read and write. The overall patients’ knowledge was 63.4%. Better knowledge was observed concerning timing (78.4%) and site of insulin injection (89.4%), while knowledge on the angle of inclination during insulin administration (43.3%) and complications of insulin therapy (49%) were low. Patients who were male gender, never married, government or NGO employees, urban residents, who completed elementary and higher education had a higher knowledge than their comparators. The majority (62%) of the study patients had a favorable attitude on insulin self-administration. Although the majority 177(72.2%) of the study patients have administered insulin themselves, only 120(49.0%) of the patients injected insulin appropriately at 45^0^. Frequent repetition of the injection site was practiced among 176(71.8%) patients and 139(56.7%) injected insulin before or immediately after food intake.

**Conclusion:**

Patients’ knowledge and attitude seem suboptimal and malpractice of insulin self-administration was reported. Therefore, the gaps should be addressed through patient education and demonstration of insulin injection during each hospital visit.

## Introduction

Diabetes mellitus (DM) is a metabolic disorder characterized by chronic hyperglycemia with impaired carbohydrate, fat and protein metabolism and resulted from either inadequate insulin secretion, resistance to the action of insulin or both [1,2].

According to the International Diabetes Federation’s report 382 million had diabetes in the year 2013 and it is estimated to reach 592 million in the year 2035. Similarly, there were more than 1.8 million diabetes patients in Ethiopia with a national prevalence of 4.36% among the adult population [3]. DM is considered the leading cause of death in most developing nations [4,5]. This might be attributed to poorly controlled hyperglycemia which is associated with several life-threatening complications such as renal failure and cardiovascular diseases [6]. Optimal glycemic control is mandatory to reduce morbidity and mortality of DM through the prevention and/or delay of complications [7]. Optimum glycemic control can be only achieved when the patients are adherent to self-management behaviors such as healthy diet, physical activity, monitoring of blood glucose, taking medications appropriately, ability to resolve diabetes problems, and healthy coping [7–11].

Insulin therapy is an essential component of medications used in DM treatment and the cornerstone of treatment in type 1 and type 2 diabetes. Despite this, at least one-third of patients fail to take their insulin as prescribed and 20% of adults deliberately miss their doses [12]. Insulin therapy presents many challenges due to complexities associated with its intricate use. Sufficient knowledge of its use can help to prevent complications, adverse patient outcomes, poor adherence to therapy and invariably poor glycemic control [13]. However, the knowledge and practice scores of patients with diabetes mellitus were not satisfactory [14]. Educating patients on self-administration of insulin helps to build self-confidence and pride of contribution in their management [15]. Moreover, an appropriate injection technique is important for proper delivery to subcutaneous tissues and to prevent intramuscular injuries and lipohypertrophy [16]. The American Diabetic Association formulated a set of guidelines for insulin storage, mixing of insulin, proper use of insulin syringe and other considerations [17]. However, patients especially in developing countries may not follow the guideline due to low socioeconomic problems.

Although insulin is recognized as the ideal treatment for DM lack of knowledge and coordination among the physicians and patients regarding appropriate insulin use is reported [18,19]. In addition to this, several studies showed that insulin injection practices were not up to the desired standard [20–22]. There is limited study in Ethiopia which focused on the knowledge, attitude and practice of insulin administration among patients with DM [23,24]. The existed studies were not in Addis Ababa the capital city of Ethiopia, which includes only type 1 diabetic patients and is limited to a specific population. Therefore, this study aimed to assess knowledge, attitude and practice of diabetic patients regarding insulin self-administration at Zewditu Memorial Hospital (ZMH) Addis Ababa, Ethiopia.

## Methods

### Study setting

The study was conducted at ZMH which is one of the state-owned public hospitals in the capital city of Ethiopia. The hospital provides comprehensive medical services with more than 400 medical staffs.

### Study design

Institution-based cross-sectional study was carried out by using interviewer-administered questionnaires to assess knowledge, attitude and practice of insulin self-administration among patients with DM. The study was conducted from February 25 to April 20, 2018.

### Study variables

Socio-demographic factors (age, sex, marital and educational status, religion, place of residence, ethnic group and occupation) and duration of DM diagnosis were the independent variables. Patients’ knowledge of insulin self-administration was the dependent variable. Besides, patients’ attitude and practice towards insulin self-administration were assessed.

### Study population and sampling procedures

The source populations were all patients with type 1 or type 2 DM, who had a follow-up at ZMH. Patients who were 18 years and above, currently taking insulin therapy and willing to participate in the study were included. Patients with a mental disorder, unable to hear and/or speak, and very sick were excluded from the study. A total of 245 patients were included in the study and systematic random sampling was employed to select the study participants.

### Data collection process

The data was collected using an interviewer-administered structured questionnaire. The data was collected by three pharmacists under the supervision of a senior clinical pharmacist. One day of training was given to the data collectors regarding the objectives of the study and how to interview the study participants. The questionnaire was developed based on previous studies [23–25] with minor modifications. The questionnaire has four parts (socio-demographic, knowledge, attitude and practice with 9, 13, 5 and 6 structured questions respectively). The knowledge part was Yes or No questions that assess the general information on diabetes mellitus and insulin self-administration.

It was first developed in English and then translated into Amharic then translated back into English by a different person to check its consistency. The questionnaire was pretested on 5% of the sample size before the actual study and appropriate correction was taken accordingly.

### Data processing and analysis

The collected data were coded, entered and analyzed by using Statistical Package for Social Sciences (SPSS) version 20 software. Descriptive statistics such as frequency distribution and percentages were performed to summarize the result. Multivariable binary logistic regression analysis was used to assess the association of the independent variables with patients’ knowledge about insulin self-administration after univariable analysis (*p*<0.2) to control confounders and *p*-value < 0.05 was considered as statistically significant.

### Ethical considerations

The study was approved by the Institutional Review Board of School of Pharmacy, College of Health Sciences, Addis Ababa University and written informed consent was obtained from the study participants.

### Operational definitions

#### Good knowledge

A patient who answered 9–13(**≥** 69.2%) correct responses from the 13 questions used to assess patients’ knowledge.

#### Average knowledge

A patient who answered 5–8 (38.5% - 61.5%) correct responses.

#### Poor knowledge

A patient who answered 0–4 (≤ 30.8%) correct responses.

#### Favorable attitude

A patient who answered 3 (60%) positive responses from the 5 questions used to assess patients’ attitude.

#### Practice

Was assessed by using six questions that explore participants’ experience with insulin utilization.

## Results

### Socio-demographic data

Among 245 patients enrolled, 132(53.9%) were male. The mean age of the patients was 53.26 ±13.43 years and majority 128(52.2%) of the patients were married. More than105 (83.7%) of the participants can at least read and write and 53(21.6%) attended higher education. The majority 219(89.4%) of the patients were urban residents and about half were married and live with diabetes mellitus for 6–10 years (Table 1).

**Table 1:** Socio-demographic data of diabetic patients taking insulin therapy in Zewditu Memorial Hospital1, 2018.

**Variable**		**Frequency (%)**
**Sex**	Male	132(53.9)
	Female	113(46.1)
**Age (years)**	Below 30	12(4.8)
	30–55	164(67.2)
	Above 55	69(28.3)
**Religion**	Orthodox Christian	114(58.8)
	Protestant	34(13.9)
	Muslim	41(16.7)
	Catholic	22(9)
	Adventist	4(1.6)
**Educational level**	No formal education	40(16.3)
	Can read and write	46(18.8)
	Primary level	25(10.2)
	Secondary level	81(33.1)
	Higher education	53(21.6)
**Occupation**	Housewife	79(32.2)
	Farmer	15(6.1)
	Government inquiry	52(21.2)
	NGO employ	39(15.9)
	Private business	60(24.5)
**Residence**	Urban	219(89.4)
	Rural	26(10.6)
**Ethnic group**	Oromo	80(32.7)
	Amhara	91(37.1)
	Tigray	39(15.9)
	Gurage	23(9.4)
	Other	12(4.9)
**Marital status**	Never married	33(13.5)
	Married	128(52.2)
	Widowed	41(16.7)
	Divorced	43(17.5)
**Duration Of Diabetes mellitus**	<5 years	37(15.1)
	6–10 Years	120(49.0)
	>10 Years	88(35.9)

NGO: Non-governmental organization.

### Knowledge towards self-administration of insulin and management of diabetes mellitus

The mean score of patients’ knowledge was 8.24±3.5 out of 13 questions used to measure their knowledge which results (63.4%). The majority 132(53.9%) of the patients had good knowledge, while 73(29.8%) and 40(16.3%) had average and poor knowledge respectively. Better knowledge was obtained regarding the timing of insulin injection (78.4%) and site of injection (89.4%). However, patients had relatively lower knowledge concerning the angle of inclination during insulin administration (43.3%), complications of insulin therapy (49%), ways to reduce pain during insulin injection (50.6%) and the impact of massage at site injection (52.2%). Moreover, 93(38.0%) patients wrongly answered that diabetes mellitus means high blood sugar (Table 2).

**Table 2:** Knowledge patients regarding diabetes mellitus and insulin therapy in Zewditu Memorial Hospital1, 2018.

**Knowledge Assessment Variables**	**Yes N(%)**	**No N(%)**
Know about diabetes mellitus	190(77.6)	55(22.4)
Diabetes mellitus means high blood sugar	152(62.0)	93(38.0)
Know about insulin	177(72.2)	68(27.8)
Insulin vial is stored in the refrigerator or cold place	174(71.0)	71(29.0)
Insulin injection is taken soon after or just before taking food	192(78.4)	53(21.6)
The sites for insulin injection are abdomen, thigh, glutei and deltoid	219(89.4)	26(10.6)
The angle to administer insulin is 45^0^	106(43.3)	139(56.7)
The distance to rotate on the same site is one thumb	150(61.2)	95(38.8)
Ways to reduce pain during insulin injection are inters the skin, do not manipulate the needle once inserted, avoiding reusing of the same site	124(50.6)	121(49.4)
The complications of insulin therapy are low blood sugar, insulin resistance and wasting of subcutaneous tissue	120(49.0)	125(51.0)
The use of rotation of the injection site is to reduce pain, prevent wasting of subcutaneous tissues	155(63.3)	90(36.7)
Massage after injection is used to enhances the rapid absorption of insulin	128(52.2)	117(47.8)
The benefit of insulin self-administration are, time saving, inexpensive and easy to take on self while traveling	189(77.1)	56(22.9)

### Factors associated with knowledge of the patients

For the purpose of data analysis, the three categories of knowledge were dichotomized and thereby good knowledge is taken as adequate, while average and poor knowledge as inadequate knowledge. In the multivariable logistic regression sex, marital status, occupation, area of residence and educational status were associated with patients’ knowledge. Patients who were male gender (AOR = 1.52, 95% CI (1.12–3.39)), never married (AOR = 3.21, 95% CI (1.72–9.69)), government employee (AOR = 2.87, 95% CI (1.08–6.31)), NGO employee (AOR = 2.55, 95% CI (1.67–8.42)) and urban residence (AOR = 2.25, 95% CI (1.18–9.51)) elementary education (AOR = 3.25, 95% CI (1.68–12.71)) and higher education AOR = 4. 35, 95% CI (1.41–10.22)) had a higher knowledge than their comparators (Table 3).

**Table 3:** Factors associated with knowledge of diabetic patients at Zewditu Memorial Hospital, 2018.

**Variables**	**Category**	**Knowledge**		**AOR**	**P-value**
		Inadequate N(%)	Adequate N(%)		
**Sex**	Male	52 (38.6)	81 (61.4)	1.52(1.12–3.39)	0.047*
	Female	62 (54.8)	51 (45.2)	1.00	
Marital Status	Never married	7 (21.2)	26 (78.8)	3.21(1.72–9.69)	0.011*
	Married	60 (46.9)	68 (53.1)	1.13(0.59–2.56)	0.261
	Widowed	25 (61.0)	16 (39.0)	0.64(0.21–0.87)	0.048*
	Divorced	21 (48.9)	22 (51.1)	1.00	
**Occupation**	House wife	42 (53.1)	37 (46.9)	1.00	
	Farmer	11(73.3)	4 (26.7)	0.45(0.25–0.89)	0.040*
	Government employ	14 (26.9)	38 (73.1)	2.87(1.08–6.31)	0.042*
	NGO employ	10 (25.7)	29 (74.3)	2.55(1.67–8.42)	0.038*
	Private business	36 (60.0)	24 (40.0)	0.95(0.28–2.20)	0.792
**Residence**	Rural	17 (65.4)	9 (34.6)	1.00	
	Urban	96 (43.9)	123 (56.1)	2.25(1.18–9.51)	0.021*
**Education Status**	No formal education	26 (65.0)	14 (35.0)	1.00	
	Can read and write	25 (54.3)	21 (45.7)	1.51(0.81–5.39)	0.188
	Primary level	8 (32.0)	17 (68.0)	3.25(1.68–12.71)	0.029*
	Secondary level	39 (48.1)	42 (51.9)	1.70(0.92–5.09)	0.077
	Higher education	15(28.3)	38 (71.7)	4.35(1.41–10.22)	0.008*

*Statistically significant (P<0.05), AOR: Adjusted odds ratio.

### Attitude of the study patients towards insulin self-administration

Patients’ attitude towards insulin therapy was assessed by using 5 questions that study their behaviors. The majority (62%) of the study patients had a favorable attitude. Three-fourth of the patients agreed that insulin self-administration was beneficial and only 55(22.4) of them believed that insulin causes other health problems. Moreover, the majority 180(73.5) of the patients disagreed that insulin self-administration is tiresome (Table 4).

**Table 4:** Attitude patients towards insulin self-administration in Zewditu Memorial Hospital, 2018.

**Attitude assessment variables**	**Agree N(%)**	**Disagree N(%)**	**Neutral N(%)**
Insulin causes other health problems	55(22.4)	108(44.1)	82(33.5)
Insulin self-administration decreases blood glucose	147(60.0)	34(13.9)	64(26.1)
Insulin self-administration is not tiresome	180(73.5)	32(13.1)	33(13.5)
Insulin self-administration does not brings stigma	141(57.6)	51(20.8)	53(21.6)
Insulin self-administration is beneficiary	185(75.5)	32(13.1)	28(11.4)

### Practice of the study patients on insulin therapy

Although the majority 177(72.2%) of the study patients administered insulin themselves, only 120(49.0%) administered insulin appropriately at 45^0^. Frequent repetition of injection site was practiced among 176(71.8%) patients and 139(56.7%) of them injected insulin before or immediately after food intake (Table 5).

**Table 5:** Practice of the study patients on insulin therapy and self-administration in Zewditu Memorial Hospital, 2018.

**Attitude patients’ practice variables**	**Yes N(%)**	**No N(%)**
Can you inject yourself in correct position?	177(72.2)	68(27.8)
Do you inject yourself with needle at 45°?	120(49.0)	125(51.0)
Do you store insulin vials in refrigerator or cold place?	228(93.1)	17(6.9)
Do you frequently repeat injection sites?	176(71.8)	69(28.9)
Do you inject insulin before or immediately after food intake?	139(56.7)	106(43.3)
Do you inject insulin into abdomen, thigh, gluteus or deltoid?	208(84.9)	37(15.1)

## Discussion

Insulin is commonly used in the management of both type 1 and type 2 diabetes mellitus. However, inadequate knowledge and malpractice on insulin self-administration could result in poor treatment outcome and insulin-related complications like hypoglycemia. Therefore, this study was aimed to assess knowledge, attitude and practice of insulin self-administration and the associated factors among diabetic patients. The overall knowledge of the study patients was 63.4% (mean score of 8.24±3.5 out of 13) which is in line with a study conducted in India (68%) [26]. However, the result was higher than the finding from Mekelle referral hospital, Ethiopia (54.4%) [23] and lower than the finding from Bangalore in India (86.7%) [27]. The discrepancies could be attributed to differences in literacy level, access to optimal education and demonstration of insulin self-administration by health care providers.

This study revealed that patients had inadequate knowledge concerning the angle of inclination during insulin administration, complications of insulin therapy, ways to reduce pain during insulin injection and the impact of massage at the site of injection. This could affect the expected treatment outcomes from insulin therapy through different ways including side effects and affect patients’ medication adherence. Hence, optimal counseling of possible outcomes of insulin therapy and demonstration of insulin administration should be provided for patients. More than half 62.0% of the patients correctly answered that diabetes mellitus means high blood sugar which was better than a similar study done in Fellegehiwot hospital in Ethiopia 33.4% [28]. The differences might be due to socio-demographic variations but further effort should be applied to improve basic information of the disease.

Associated factors of patients’ knowledge were identified to find possible strategies to improve their knowledge. In the present study sex, marital status, occupation, area of residence and educational status had a statistically significant association with patients’ knowledge. Patients who were never married, government and NGO employee had a higher knowledge by 3.21, 2.87 and 2.55 respectively as compared to their comparators. Knowledge of farmer patients was lower than the comparators by 1.55 times. A similar finding was reported in a study conducted in Hawasa referral hospital, Ethiopia [24]. Never married patients could be the young patients who have relatively better educational status and probably with a high rate of type 1 DM that could improve their knowledge. Government and NGO employees are often educated peoples and may have better access to information as well as better understanding.

Similarly, urban residents, patients who completed elementary education and higher education had a higher knowledge than their comparators by 2.25, 3.25 and 4.35 respectively. A similar finding was reported in a study conducted in Hawasa referral hospital [24]. The reason could be due to patients who had at least completed primary education may have a better chance of exposure to different communication aids like leaflets, magazines and books. In addition, they may have few barriers in communicating with the health care team besides their potential to grasp information’s already communicated [29].

The overall favorable attitude towards insulin self-administration was 62% which was lower than a study done in Bangalore.in India (81.7%) [27]. But, the present finding was higher than the other finding in India (32%) [26]. This difference might be due to socio-cultural, health literacy and access to health promotion regarding insulin therapy. A significant number of patients 51(20.8%) had a negative attitude that insulin self-administration brings stigma. This could cause suboptimal insulin utilization behavior that will affect sugar control. Hence, health care providers should focus on promoting health education and awareness creation regarding insulin use.

In the present study, the majority 177(72.2%) of the study patients administered insulin by themselves and the rest 21.8% were dependent on others for the administration of their medication. This could also affect medication adherence and patients should be encouraged as much as possible for self-administration with clear demonstration and counseling. About half of the patients did not inject insulin appropriately at 45^0^ and 71.8% of patients frequently injected at the same site of injection. This might cause unwanted effects like pain and lipoatrophy at the injection site. Furthermore, only 56.7% of the study patients injected insulin before or immediately after food intake and the rest were administering insulin regardless of food including during fasting. This is a common problem and a major cause of insulin-induced hypoglycemia. Therefore patients taking insulin should be counseled and followed for appropriate insulin administration in each hospital visit.

### Limitation of the study

This study was conducted in a single institution that might limit its generalizability. Duration of insulin use, that could have an association with knowledge and practice of insulin self- administration, was not collected in the present study. Besides, the practice of insulin self-administration was assessed solely on patients’ responses without actual observation that could underestimate the magnitude of malpractice.

## Conclusion

The overall knowledge of the study patients regarding insulin self-administration was suboptimal especially related to the angle of inclination during insulin administration, complications of insulin therapy, ways to reduce pain during insulin injection and impact of massage at site injection. Hence, adequate patient education should be addressed to fill the knowledge gaps and improve insulin therapy outcomes. Furthermore, sex, marital status, occupation, area of residence and educational status were associated factors of patients’ knowledge and possible strategies should be sought to act on those patients accordingly. Patients’ attitude on insulin therapy was also inadequate that requires patient education and awareness creation. Despite the vast majority of the study patients administered insulin for themselves, a significant number of malpractices were found. Therefore, the gaps should be addressed with an appropriate demonstration of insulin injection.

## Supporting information

S1 File Data collection tool. (DOCX)

## Abbreviations

DM: Diabetes Mellitus
NGO: Nongovernmental organization
ZMH: Zewditu Memorial Hospital
