# Supplementary material for: Knowledge, attitude and practice towards insulin self-administration and associated factors among diabetic patients at Zewditu Memorial Hospital, Ethiopia
Source: PLoS One. 2021 Feb 8;16(2):e0246741. doi: 10.1371/journal.pone.0246741 (PMC7870072; doi:10.1371/journal.pone.0246741)
Supplement: S1 File — (DOCX) [file pone.0246741.s001.docx]

**Data collection toll**

**English version**

1. **Socio-demographic characteristics**
2. Sex: □ Male □ Female
3. Age: □ Below 30 years □ 30-55 years □ Above 55 years
4. Religion: □ Orthodox Christian □ Protestant □ Muslim

□ Catholic □ Adventist

1. Educational level: □ No formal education □ Can read and write

□ Primary level □ Secondary level □ Higher education

1. Occupation: □ House wife □ Farmer □ Government inquiry

□ NGO employ □ Private business

1. Residence: □ Urban □ Rural
2. Ethnic group: □ Oromo □ Amhara □ Tigray □ Gurage □ Other
3. Marital status: □ Never married □ Married □ Widowed □ Divorced
4. Duration of Diabetes mellitus: □ <5 years □ 6-10 Years □ >10 Years
5. **Items related to patients knowledge**

| **Descriptions** | Yes | No |
| --- | --- | --- |
| Know about diabetes mellitus | □ | □ |
| Diabetes mellitus means high blood sugar | □ | □ |
| Know about insulin | □ | □ |
| Insulin vial is stored in the refrigerator or cold place | □ | □ |
| Insulin injection is taken soon after or just before taking food | □ | □ |
| The sites for insulin injection are abdomen, thigh, glutei and deltoid | □ | □ |
| The angle to administer insulin is 45^0^ | □ | □ |
| The distance to rotate on the same site is one thumb | □ | □ |
| Ways to reduce pain during insulin injection are inters the skin, do not manipulate the needle once inserted, avoiding re using of the same site | □ | □ |
| The complications of insulin therapy are low blood sugar, insulin resistance and wasting of subcutaneous tissue | □ | □ |
| The use of rotation of the injection site is to reduce pain, prevent wasting of subcutaneous tissues | □ | □ |
| Massage after injection is used to enhances the rapid absorption of insulin | □ | □ |
| The benefit of insulin self-administration are, time saving, inexpensive and easy to take on self while traveling | □ | □ |

1. **Items related to patients attitude**

| **Descriptions** | Agree | Disagree | neutral |
| --- | --- | --- | --- |
| Insulin causes other health problems | □ | □ | □ |
| Insulin self-administration decreases blood glucose | □ | □ | □ |
| Insulin self-administration is not tiresome | □ | □ | □ |
| Insulin self-administration does not brings stigma | □ | □ | □ |
| Insulin self-administration is beneficiary | □ | □ | □ |

1. **Items related to Practice**

| Questions | Yes | No |
| --- | --- | --- |
| Can you inject yourself in correct position? | □ | □ |
| Do you inject yourself with needle at 45°? | □ | □ |
| Do you store insulin vials in refrigerator or cold place? | □ | □ |
| Do you frequently repeat injection sites? | □ | □ |
| Do you inject insulin before or immediately after food intake? | □ | □ |
| Do you inject insulin into abdomen, thigh, gluteus or deltoid? | □ | □ |

**Amharic version**

1. **ማህበራዊና አካባቢያዊ ምረጃዎች**
2. ጾታ: □ ወንድ □ ሴት
3. እድሜ : □ <30 አመት □ 30-55 አመት □ >55 አመት
4. ሀይማኖት: □ ኦርቶዶክስ □ ጴንጤ □ ሙስሊም □ ካቶሊክ □ አድቬንቲስት
5. የትምህርት ደረጃ : □ መጽሃፍ እና ማንበብ የማይችል □ መጽሃፍ እና ማንበብ የሚችል

□ አንደኛ ደረጃ □ ሁለተኛ ደረጃ □ ከፍተኛ ደረጃ

1. ስራ: □ የቤት እመቤት □ ገበሬ □ የመንግስት ሰራተኛ

□ መንግስታዊ ያልሆነ ድርጅት ሰራተኛ □ ነጋዴ

1. መኖሪያ : □ ከተማ □ ገጠር
2. ጎሳ ወይም ብሄር: □ ኦሮሞ □ አማራ □ ትግሬ □ ጉራጌ □ ሌላ
3. የጋብቻ ሁኔታ: □ ያላገባ □ ያገባ □ የሞተበት □ የተፋታ
4. ከስኳር ህመም ጋር የቆዩበት ጊዜ : □ <5 አመት □ 6-10 አመት □ >10 አመት
5. **ከእውቀት ጋር የተያያዙ መጠይቆች**

| **መጠይቆች** | አዎ | አይደለም |
| --- | --- | --- |
| ስለ ስኳር ህመም ምንነት አውቃለው | □ | □ |
| የስኳር ህመም የሚባለው ደም ውስጥ ያለው ስኳር ከፍ ሲል ነው | □ | □ |
| ስለ ኢንሱልን ምንነት አውቃለው | □ | □ |
| የኢንሱልን ብልቃጥ ፍሪጅ ውስጥ ወይም ቀዝቃዛ ቦታ ላይ ይቀመጣል | □ | □ |
| የኢንሱልን መርፌ ልክ ምግብ እንደተበላ ወይም ከምግብ በፊት ይወሰዳል | □ | □ |
| የኢንሱሊን መርፌ መወጊያ ቦታዎች ሆድ ፣ ታፋ፣ ትኬሻ ጡንቻ ናቸው | □ | □ |
| ኢንሱሊን የሚሰጠው በ 45 ^0^ ማዕዘን ነው | □ | □ |
| በሁለት ተመሳሳይ መርፌ የሚስጥባቸው ቦታዎች ማሽከርከሪያ ርቅት አንድ አውራ ጣት ያክል ነው | □ | □ |
| የኢንሱሊን መርፌ በምንወጋበት ጊዜ ህመም ለመቀነስ መርፌዉን አለማንቀሳቀስ እና ተመሳሳይ ቦታዎችን አለመጠቀም ነው | □ | □ |
| የኢንሱሊን መጠቀም የጎኒዎሽ ችግር የሚባሉት ከልክ በላይ ደም ውስጥ ያለው ስኳር መቀነስ ፣ የኢንሱሊን መላመድ እና ቆዳ ውስጥ ያሉ ህብረ-ህዋሳትን ማዳከም ናቸው | □ | □ |
| የመርፌ መወጊያ ቦታዎችን የምናቀያይረው ህመም ለመቀነስ እና ቆዳ ውስጥ ያሉ ህብረ-ህዋሳትን ማዳከም ለመከላክል ነው | □ | □ |
| መርፌ ከሰጠን በኋላ ቦታውን የምናሸው ኢንሱልን ደም ውስጥ የሚደርስበትን ፍጥነት ለመጨመር ነው | □ | □ |
| ኢንሱሊንን ራስ መወጋት ጊዜ ይቆጥባል ፡ ርካሽ እና እንዲሁም ጉዞ ላይ ለመውሰድ ይቀላል | □ | □ |

1. **ከስነ-ፀባይ የተያያዙ መጠይቆች**

| **መጠይቆች** | እስማማለው | አልስማማም | ገለልተኛ |
| --- | --- | --- | --- |
| ኢንሱሊን ሌላ የጤና ችግሮችን ያመጣል | □ | □ | □ |
| ኢንሱሊንን ራስ መወጋት ደም ዉስጥ ያለውን ስኳር መጠን ይቀንሳል | □ | □ | □ |
| ኢንሱሊንን ራስ መወጋት አሰልቺ ወይም አድካሚ አይደለም | □ | □ | □ |
| ኢንሱሊንን ራስ መወጋት መገለል አያመጣም | □ | □ | □ |
| ኢንሱሊንን ራስ መወጋት ጠቃሚ ነው | □ | □ | □ |

1. **ከተግባር ጋር የተያያዙ መጠይቆች**

| **መጠይቆች** | አዎ | አይደለም |
| --- | --- | --- |
| በራስዎን ትክክለኛውን መርፌ ቦታ መውጋት ይችላሉ? | □ | □ |
| መርፌውን በ45° ላይ ይወጋሉን? | □ | □ |
| የኢንሱሊንን ብልቃጥ ፍሪጅ ውስጥ ያስቀምጣሉ? | □ | □ |
| አዘውትረው መርፌ መውጊያ ቦታን ደጋግመው ይጠቀማሉ? | □ | □ |
| ኢንሱሊንን ከምግብ በፊት ወይም ምግብ እንደወሰዱ ወዲያውኑ ይወጋሉ? | □ | □ |
| ኢንሱሊንን ሆድ ፣ ታፋ፣ ትኬሻ ጡንቻ ላይ ይወጋሉ? | □ | □ |
